# Editorial: Trabectedin, lurbinectedin, and other marine-derived anticancer alkaloids on solid cancer: Mechanisms of action, clinical impact, and future perspectives

**DOI:** 10.3389/fonc.2022.1115342

**Published:** 2023-01-25

**Authors:** Alberto Zambelli, Maurizio D’Incalci

**Affiliations:** ^1^ Department of Biomedical Sciences, Humanitas University, Milan, Italy; ^2^ Medical Oncology and Hematology Unit, Humanitas Cancer Center, IRCCS Humanitas Research Hospital, Milan, Italy

**Keywords:** marine drugs, trabectedin, lurbinectedin, eribulin and related compounds, solid tumor

The growing knowledge of the molecular pathogenesis of many diseases allows designing drugs able to bind or inhibit specific molecular targets with high specificity. As far as cancer, this approach has been very successful and several drugs eventually have been developed as inhibitors of oncogenes and antagonist of aberrantly deregulated pathways, in specific human neoplasms. The Hallmarks of cancer - recently overviewed by Hanahan - are multiple and complex (1). They involve cell proliferation signaling, evading cell-growth suppressors, resisting cell death, enabling replicative immortality, inducing neo-angiogenesis, activating invasion and metastases, reprogramming of cell metabolism and avoiding anti-cancer immune engagement.

The explosion of knowledge on cancer biology has offered tremendous opportunities to develop novel synthetic compounds or antibodies acting by inhibiting different cancer targets. However, the results achieved with these target therapies have been frequently less impressive than expected, deriving only in few cases a meaningful improvement in patients’ overall survival (2). This is probably due to the fact that for some human malignancies no drugable driving-targets have been eventually identified, whereas for others rapid occurrence of resistance is observed because of genomic instability and high biological heterogeneity, as crucial features of most human advanced malignancies.

In this context, the identification of new natural products with antitumor activity is far from being an obsolete strategy and in particular marine chemistry provides access to a large number of compounds with unique chemical structure and potentially exploitable biological and pharmacological properties. Indeed, the exploration of the seas with myriads of microenvironments has played an important role in our understanding of the adaptation of life to hostile environment, mainly trough the production of compounds that in some cases have turned out to be useful drugs in the fight against cancer.

In this special issue entitled *“Trabectedin, Lurbinectedin, and other Marine-Derived Anticancer Alkaloids”* basic scientists and clinical researchers provide some interesting examples of marine compounds that are already part of the therapeutic armamentarium of the medical oncologists (i.e. trabectedin and eribulin) or under preclinical and clinical investigation, as in the case of lurbinectedin.

Trabectedin was one of the first marine-derived anti-neoplastic drugs approved in solid tumor, in particular for the treatment of soft tissue sarcomas and relapsed platinum-sensitive ovarian cancer. Its unique mechanism of action, related to transcription regulation, DNA-repair machinery interference and direct effects on tumor microenvironment, is paving the way for new investigations in the field, as highlighted in the papers published in this Research Topic of *Frontiers in Oncology*, that reports a mixture of updated reviews and original contributions. In the paper by Merlini et al. a potential predictive biomarker of response to trabectedin combined with the PARP inhibitor olaparib is proposed in advanced bone and soft tissue sarcomas. In the paper by Allavena et al. the unique effects of trabectedin and lurbinectedin on tumor microenvironment are carefully described showing that both drugs can potentiate immunotherapy with checkpoint inhibitors by reducing the immune escape and the number and function of the immunosuppressant tumor associated macrophages (TAM). Namely, lurbinectedin shares some mechanistic properties of trabectedin but has also some distinct pharmacokinetic/pharmacodynamic features that are relevant for the encouraging toxicity profile and large spectrum of activity, as overviewed in the papers by Gadducci and Cosio, and by Musacchio et al. this latter mainly focusing on ovarian cancer. The paper by Heredia-Soto et al. shows the marine antimitotic agent Plocabulin has a preclinical potent anti-proliferative activity and migration in several ovarian cancer cell lines. Although the data presented were preliminary and no conclusion can be definitively drawn on the therapeutic index of Plocabulin, nevertheless the drug appears to be equally effective in platinum sensitive/resistant ovarian cancer cells, a finding of potential clinical relevance.

The papers by Nakamura and Sudo and by Phillips et al. provide updated overviews on the activity of trabectedin and eribulin in different histotypes of soft tissue sarcomas. The paper by Sanctis et al. highlights the therapeutic activity of several marine anticancer drugs - including trabectedin, lurbinectedin and eribulin - in breast cancer. In addition, the paper overviews the recent data obtained with the very potent marine compound monomethyl auristatin bound to specific antibodies, with high selectivity for breast cancer. As illustrated, these novel antibody-drug conjugates (ADC) show promising activity in breast cancer and likely in others cancer types.

Several papers of the issue report the available data on the pharmacological properties and specific toxicities of marine natural products, such as trabectedin and eribulin that have been used in the clinic for a long. In particular, the paper by Keritam et al. focuses on the toxicity associated to the extravasation of trabectedin and provides preclinical original data to assess this type of undesired effects.

Certainly, the papers published on this issue can be an important source of references for researchers and clinical oncologists and may fuel further interest in marine products that we feel will be not only important for relevant biological discoveries but will also contribute to improve the therapy of many human tumors, in the current era of molecular medicine.

## Author contributions

AZ and MD equally contributed to the manuscript conceptualization, writing, review, and editing. All authors contributed to the article and approved the submitted version.
